# What We Owe to Experiments on Animals

**Published:** 1903-12-12

**Authors:** Stephen Paget


### Hospital Clinics and Medical Progress.

### WHAT WE OWE TO EXPERIMENTS ON ANIMALS.

By Stephen Paget.

*I—PHYSIOLOGY.*

(*Continued from page* 166.)

### III.—The Gastric Juice.

At the end of the seventeenth century, there was a long controversy at Florence between Borelli and Valisnieri. Borelli held that the digestion of food in the stomach was a mere grinding and breaking-up of the food, like the action of a bird's gizzard: Valisnieri, who had found an acid fluid in the stomach of an ostrich, said that this fluid was the active principle of digestion, a sort of *aqua fortis.* These opposed views were the starting-point of Réaumur's work in 1752. He made birds swallow food enclosed in small tubes with holes bored in them, so that the food was kept from mechanical pressure but was exposed to chemical action: in other experiments, he made them swallow bits of sponge attached to threads, which he withdrew, and tested the fluid thus obtained. Spallanzani (1783) made similar observations on carnivorous animals. All later work on the subject was founded on this work. But, of course, results came very slowly, and out of much uncertainty, for this reason, that physiology could not run ahead of organic chemistry. In 1823 the French Academy proposed digestion as the subject for a prize, and divided the prize between the work of Tiedemann and Gmelin (Germany) and Leuret and Lassaigne (France). In 1834 Eberlé published his observations on the extraction of the gastric juice from the mucous membrane of the stomach after death: this method gave rise to the manufacture of pepsin. In 1842 Blondlot studied the gastric juice by means of an artificial fistula. In later years, experiments have been made on the movements of the stomach, on the relations between digestion and the nervous system, and on other problems of nutrition and absorption which were beyond the range of the older physiologists.

Reference must be made here to the well known case of Alexis St. Martin. He was a young Canadian, a traveller for an American fur company; he was shot in the stomach, on June 6th, 1822, and recovered, but with a permanent fistula communicating with his stomach. No operation was done to close it; and in 1825 Dr. William Beaumont, a surgeon in the American Army, took the young man into his service, and made experiments on him during eight years. "During the whole of these periods," says Dr. Beaumont, "from the spring of 1824 to the present time (1833), he has enjoyed general good health, and perhaps suffered much less predisposition to disease than is common to men of his age and circumstances in life. He has been active, athletic, and vigorous; exercising, eating, and drinking like other healthy and active people. For the last four months he has been unusually plethoric and robust, though constantly subjected to a continuous series of experiments on the interior of the stomach; allowing to be introduced or taken out at the aperture different kinds of food, drinks, elastic catheters, thermometer tubes, gastric juice, chyme, etc., almost daily, and sometimes hourly."

Two things are to be noted about this case. First, it came too late to lead to any great discovery: as Dr. Beaumont says, "I make no claim to originality in my opinions as it respects the existence and operation of the gastric juice. My experiments confirm the doctrines (with some modifications) taught by Spallanzani, and many of the most enlightened physiological writers." Next, it disproves the statement often made, that an animal with an artificial fistula is kept in pain.

### IV.—Glycogen.

Claude Bernard, in 1853, published his book, "A New Function of the Liver," in which he tells the story of his discovery that glycogen is produced in the liver. Long before him, men knew that diabetes was attended by an increase of sugar in the body; but this clinical fact got no help from physiology till Bernard took it up. He says, "My first researches into the assimilation and destruction of sugar in the living organism were made in 1843, and in my inaugural thesis (December, 1843) I published my first experiments on the subject. I succeeded in demonstrating a fact hitherto unknown, that cane-sugar cannot be directly destroyed in the blood. . . . I had soon to give up my first point of view, because this question of the existence of a sugar-producing organ, that I had thought such a hard problem of physiology, was really the first thing revealed to me, of itself, as it were, at once." He tells how he kept two dogs on different diets, one with sugar, the other without it; then killed them during digestion, and to his great surprise found sugar in the blood of the veins coming from the liver, in both dogs alike. And he goes on to say, "At last, after many attempts and many false ideas that I had to put right by feeling my way, I succeeded in showing that in dogs fed on meat the blood passing through the portal vein does not contain sugar before it reaches the liver; but when it leaves the liver and comes by the hepatic veins into the inferior vena cava, this same blood contains a considerable quantity of a sugary substance."

Sir Michael Foster says of Bernard's work in this field of physiology:—"As a mere contribution to the history of sugar within the animal body, as a link in the chain of special problems connected with digestion and nutrition, its value was very great. Even greater, perhaps, was its effect as a contribution to general views. The view that the animal body, in contrast to the plant, could not construct, could only destroy, was already being shaken. But evidence, however strong, offered in the form of numerical comparisons between income and output, failed to produce anything like the conviction which was brought home to everyone by the demonstration that a substance was actually formed within the animal body, and by the exhibition of the substance so formed.

"No less revolutionary was the demonstration that the liver had other things to do in the animal economy besides secreting bile. This, at one blow, destroyed the then dominant conception that the animal body was to be regarded as a bundle of organs, each with its appropriate function.

"No less pregnant of future discoveries was the idea suggested by this newly found out action of the hepatic tissue, the idea happily formulated by Bernard as 'internal secretion.' The study of these internal secretions constitutes a path of inquiry which has already been trod with conspicuous success, and which promises to lead to untold discoveries of the greatest moment: the gate to this path was opened by Bernard's work."

V.—The Pancreas.

Before the time of Regnier de Graaf (1660) nothing, or next to nothing, was known about the pancreas. Wirsung, a few years before, had discovered its duct, but nobody understood its influence on the general economy of the body. The first thing to be done was to observe what it secreted; and this de Graaf succeeded in doing by means of a fistula. "I put my hand to the work," he says, "and, though many times I despaired of success, yet at last, by the blessing of God on my work and prayers, in the year 1660 I discovered a way of collecting the pancreatic juice."

It is to be observed that de Graaf was a whole century earlier than Réaumur. His work was lost, because it got no help from organic chemistry; and it remained lost for nearly two centuries. Bernard, in 1846, went back to de Graaf's method of collecting the pancreatic juice. Sir Michael Foster says of his work, "Valentin, it is true, had in 1844 not only inferred that the pancreatic juice had an action on starch, but confirmed his view by actual experiment with the juice expressed from the gland; and Eberlé had suggested that the juice had some action on fat; but Bernard at one stroke made clear its threefold action. He showed that it on the one hand emulsified, and on the other hand split up into fatty acids and glycerine, the neutral fats; he clearly proved that it had a powerful action on starch, converting it into sugar; and lastly, he laid bare its remarkable action on proteid matters."

Mering and Minkowski, about 1890, discovered that the pancreas, over and above its secretion into the alimentary canal, has a further influence, an "internal secretion," which maintains the balance of the health of the body. They found that the removal of the pancreas was followed by diabetes; but this did not occur, if a portion, even a very small portion, were left in the body, either in its proper place or transplanted to some other part of the body. More recently, important observations have been made by Professor Starling as to the influence of the mucous membrane of the alimentary canal, in the neighbourhood of the pancreas, on the flow of pancreatic secretion.

VI.—The Growth of Bone.

Du Hamel (1739), taking advantage of the fact that the bones of animals fed near dye-works may become stained with the dye in their food, made experiments on young pigs, feeding them at one time on food stained with madder, at another time on unstained food. By this method, which produced alternate rings of red and white in the shafts of the bones, he proved that the periosteum has the power of forming bone out of its own substance. Later, by the help of other experiments on animals, the growth of the bones was more thoroughly studied; not only their growth in thickness, but also their growth in length, and the bearing on surgery of these physiological facts. The names of Syme, Stanley, and Ollier are associated with this part of the work.

(*To be continued.*)

